# Potential applications of pulsed electric field in the fermented wine industry

**DOI:** 10.3389/fnut.2022.1048632

**Published:** 2022-11-02

**Authors:** Yuanxin Feng, Tao Yang, Yongniu Zhang, Ailin Zhang, Lili Gai, Debao Niu

**Affiliations:** ^1^College of Light Industry and Food Engineering, Guangxi University, Nanning, China; ^2^School of Pharmacy, Hainan Medical University, Haikou, China

**Keywords:** pulsed electric field, fermented wine, mechanism, aging, application

## Abstract

Fermented wine refers to alcoholic beverages with complex flavor substances directly produced by raw materials (fruit or rice) through microbial fermentation (yeast and bacteria). Its production steps usually include saccharification, fermentation, filtration, sterilization, aging, etc., which is a complicated and time-consuming process. Pulsed electric field (PEF) is a promising non-thermal food processing technology. Researchers have made tremendous progress in the potential application of PEF in the fermented wine industry over the past few years. The objective of this paper is to systematically review the achievements of PEF technology applied to the winemaking and aging process of fermented wine. Research on the application of PEF in fermented wine suggests that PEF treatment has the following advantages: (1) shortening the maceration time of brewing materials; (2) promoting the extraction of main functional components; (3) enhancing the color of fermented wine; (4) inactivating spoilage microorganisms; and (5) accelerating the formation of aroma substances. These are mainly related to PEF-induced electroporation of biomembranes, changes in molecular structure and the occurrence of chemical reactions. In addition, the key points of PEF treatments for fermented wine are discussed and some negative impacts and research directions are proposed.

## Introduction

Fermented wine, known as original juice wine, is made of raw materials containing starch and sugar (fruit or rice) through the fermentation of yeast and bacteria to produce complex flavor substances and alcohol, such as red wine, white wine, and rice wine. The production of fermented wine is a complex and time-consuming process, including saccharification, fermentation, filtration, sterilization, and a series of continuous technological procedures. Any operation introduced in the brewing process can affect the physicochemical properties and sensory characteristics of the finished wine (1, 2). Some freshly fermented wines are not suitable for immediate drinking due to their spicy taste, pungent smell, precarious color, and aroma. In order to improve their stability, quality and sensory characteristics, these freshly fermented wines are customarily required to undergo post-processing, i.e., aging (also known as maturation) (3). However, several disadvantages of using oak barrels, pottery or other modern containers for naturally mature fermented wines, such as red wine and rice wine, are worth investigating, including long time, large storage space, and the presence of undesirable microorganisms that cause food contamination and produce unpleasant tastes (4). Therefore, it is necessary to adopt innovative technologies to optimize or accelerate the winemaking and aging of fermented wine.

Pulsed electric field (PEF) is a promising non-thermal food processing technology, which can effectively ensure the good quality of products (5–9). Recent technological developments, especially the use of continuous processing chambers, have provided more possibilities for scaling up the application of PEF technology, which has attracted widespread attention (10–12). The potential applications of PEF in the fermented wine industry have been extensively studied worldwide (13–16). Therefore, this review aims to systematically summarize the achievements of PEF technology in the fermented wine industry, and discuss the corresponding processing principles and some negative effects, so as to provide references for future research directions.

## Principles of pulsed electric field

Pulsed electric field is a promising non-thermal technology, which applies a high-intensity electric field pulse for a short time (treatment time usually in the microsecond scale) to food or raw material between two electrodes (17–19). The exponentially decaying waves and square waves are commonly used in PEF processing, and square wave pulses are considered better than the exponential decay wave pulses because the former allows the material to be treated with a sustained and constant intensity for the total duration of the pulse (5). The factors affecting the efficiency of PEF treatment include electric field intensity, pulse number, pulse shape, pulse time/length, and initial temperature of processing medium (raw material), etc. (20). Among them, electric field intensity and treatment time (*t* = pulse duration × pulse number) are the main processing factors, and increasing the intensity of these two parameters generally improves the processing efficiency of PEF (5).

**FIGURE 1:**
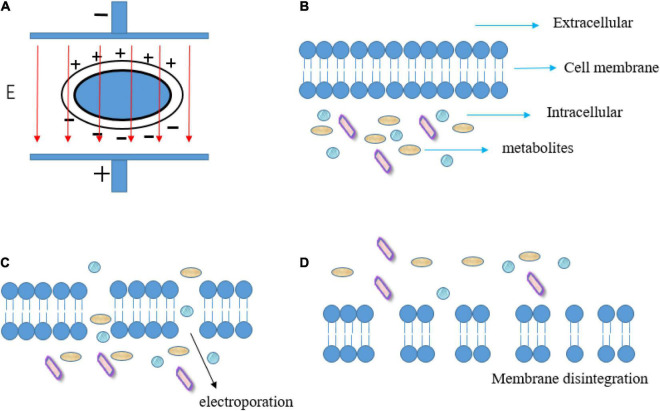
Schematic diagram of transmembrane potential induced by PEF **(A)**, membrane before electroporation **(B)**, electroporation of cell membrane **(C)**, and membrane disintegration induced by excessive PEF treatment **(D)**. Adapted from Mahesha et al. (96).

When an external electric field is applied, the charge accumulation on the membrane surface causes an increase in the transmembrane potential on both sides of the membrane. After the critical value of transmembrane potential is exceeded, the electroporation (reversible or irreversible) will be produced in cell membranes, resulting in a sharp increase in membrane permeability (21, 22). Animal and plant cells require a lower critical electric field intensity (0.5–2 kV/cm) for electroporation because their cell sizes are larger than microbial cells (10–14 kV/cm) (23). The electroporation dramatically increases membrane permeability which may affect mass transfer or cell rupture (Figure 1). Therefore, PEF technology has received extensive attention and research in a variety of food processing, such as food dehydration (24), sterilization (25), promotion of extraction (26), and reduction of pesticide residues (27). In addition, the PEF treatment promotes the ionization and polarization of molecules, changes the internal molecular arrangement, improves the effective collision rate between molecules, speeds up the chemical reaction rate in dynamic equilibrium, and reduces the activation energy required for the reaction. Hence, PEF is gradually used to accelerate the oxidation, reduction, association, hydrolysis, and other reactions in food (5, 25–28), especially liquid food such as fruit juices and alcoholic beverages (29). Meanwhile, compared with other non-thermal processes, such as high hydrostatic pressure (high equipment costs and discontinuous production) (30), the PEF technology also exhibits several advantages, such as shorter processing time, lower treatment temperature, and continuous flow processing (31).

## Applicability of pulsed electric field

### Enhancement of primordial composition extraction

The efficient extraction of functional components from target tissues or cells is a major problem prior to fermentation. The raw materials are usually pressed and macerated for a long time to extract their active ingredients such as sugar and polyphenols in traditional brewing, thus improving the yield and quality of fermented wine (32, 33). PEF is adopted as a new way for promoting the extraction of active ingredients, shortening the immersion time, and promoting alcohol fermentation. This is mainly attributed to electroporation induced by PEF treatment (Figure 1), which increases the permeability of cell membrane to ions and macromolecules, thus promoting the release of intracellular substances (18). For example, polyphenols, including phenolic acids, flavanols, flavonols, anthocyanins, and stilbenes, are the most important functional compounds in winemaking, since they have multiple biological effects and contribute to the wine’s distinctive color (34). Unfortunately, only 40% of anthocyanins and 20% of tannins from grape skins are transferred to wine in the case of traditional winemaking (35, 36). This relatively low extraction efficiency is the result of insufficient permeabilization of cell walls and cytoplasmic membranes. Hence, PEF treatment has great potential to promote the extraction of functional components in the maceration process. Interestingly, although PEF treatment can promote the dissolution of active ingredients during the maceration stage, there are other studies in which the PEF treatment of the samples did not affect some enological parameters of the grape juice samples (37). Clodoveo et al. (38) describe the mechanism that PEF treatment can destroy the cell membranes of grapes, and this treatment has no effect on alcohol content, total acidity, pH, volatile acidity and concentration in reducing sugar. It is worth noting that high voltage is usually required to produce electroporation, but high-intensity PEF treatment has a strong electrolytic effect, and its high electric field strength and long treatment time may lead to the degradation of some macromolecular substances.

#### Improvement of juice yield

The yield of fruit juice is an important index to measure the effect of maceration, and it obviously increases by PEF treatment in the brewing process. After applying 11 kJ/kg (1.5 kV/cm, 8 μs) and 22 kJ/kg (1.5 kV/cm, 16 μs) of PEF treatments, the yield of grape juice increased by 8.9 and 4.3% compared with 78.0% (w/w) of the untreated sample, respectively (14). Similarly, an increase in “Pinot Noir” grape juice yield was observed, which may be attributed to increased cell membrane permeability and accelerated mass transfer after PEF treatment (1.5 kV/cm, 1,033 pulses) (39). Meanwhile, PEF treatment (1.5 kV/cm, 1,033 pulses) of grapes increased the release rate of anthocyanins, thus achieving an adsorption-desorption equilibrium between the anthocyanins inside the skin and outside the juice within a shorter time. As a significant property, dry extract consists of fixed wine compounds, including sugars, glycerol, organic acids, phenolics, and minerals, contribute to the development of distinctive taste and in turn affect the sensory characteristics and quality of the wine (16). Compared with the control group, the dry extract content in jujube wine increased by 10% after PEF treatment (1.5 kV/cm, 50 pulses), which may be related to the improvement of mass transfer in pulp tissues, indicating that PEF pretreatment had a positive impact on jujube wine quality (Table 1). The increases in total acidity and phenolics could also explain the cause of the elevated dry extract contents, which was consistent with the previous study (1.5 kV/cm, 16 μs) (14). Skin maceration and maturation in wooden barrels have also been reported to improve dry extracts and obtain good mouth-feel properties, but require a long time-consuming (40).

**TABLE 1:** Application examples of PEF treatment for fermented wine.

Applications	Targets of treatment	Materials	Treatment conditions	Effects	References
Enhancement of extraction	Juice yield	Garganega white grapes	PEF: 1.5 kV/cm, 8 μs, and 16 μs	The yield of grape juice increased by 8.9 and 4.3% compared with 78.0% (w/w) of the control sample, respectively.	(14)
	Dry extract	Jujube wine	PEF: 1.5 kV/cm, 50 pulses	The dry extract content increased by 10%.	(16)
	Polyphenols	Pinot Noir and Merlot must	PEF: 8 kV/cm, 344 Hz, 300 s	The content of total phenols is 2 times and 1.5 times higher than the control sample, respectively.	(42)
	Polyphenols	Jujube pulp	PEF: 1.5 kV/cm, 0.1 kHz, 10 μs	The extraction rate was 45% higher than the untreated sample.	(43)
Enhancement of color	Color intensity	Cabernet Sauvignon red wine	PEF: 5 kV/cm, 122 Hz, 50 pulses, specific energy for 3.67 kJ/kg	The color intensity increased by 31% during aging in the bottle.	(48)
	Pigment	Marechal Foch grapes	PEF: 3.3 kV/cm and 5 kV/cm for 10 s, at a frequency of 20 pulses	The share of the red parameter (*a) in wines is respectively higher by 15 and 24% than those untreated samples.	(53)
	Anthocyanins	Cabernet Sauvignon rosé wines	PEF: 5 kV/cm, 50 pulses	14 anthocyanins were increased in different degrees after 2 months of storage in the bottle.	(54)
	Proanthocyanidins	Merlot red wine	PEF: 6–24 kV/cm, 10 μs, 10 Hz, 0–300 pulses	The effect was closer to aging when the field strength is below 18 kV/cm.	(60)
Inactivation of spoilage microorganisms	*Brettanomyces bruxellensis*	Cabernet Sauvignon red wine	PEF: 50 kV/cm, 100 Hz, 78 μs	>6 log reductions	(25)
	*P. pentosaceus*	Tempranillo red wine	PEF: 22 kV/cm, 154 μs, specific energy for 62 kJ/kg	1.93 log reductions	(65)
	*Saccharomyces bayanus*	Bogazkere red wine	PEF: 31 kV/cm, 3 μs, 40 ml/min of flow rate	5.4 log reductions	(68)
	*Saccharomyces cerevisiae*	Chinese rice wine	PEF: 12 kV/cm, 120 μs, 35°C,	2.88 log reductions	(77)
	*Acetobacter* sp.	–	PEF: 20 kV/cm, 6.0 ms, with 9% ethanol	The inactivation rate could reach 5.17 log reductions, significantly higher than that cultivated without ethanol (3.22 log).	(22)
Formation of aroma substances during aging	Fatty acids	Chardonnay white wine	PEF: 6 kV/cm, 10 μs, 100 pulses	The trend of tartaric acid, oxalic acid, citric acid, and succinic acid was similar to those aging in bottles.	(10)
	Free amino acids	Shaoxing Huangjiu	PEF: 2 kV/cm, 0.5 Hz, 36 pulses	The sweet and MSG-like amino acids reached the aging level, and bitter amino acids showed a declining trend.	(82)
	Polyphenols	Grenache wines	PEF: 4 kV/cm, 100 μs, specific energy for 6.2 kJ/kg	Flavan-3-ol monomers catechin and epicatechin promoted polymerization to form tannins similar to aged wines.	(13)
	Esters	–	PEF: 20 kV/cm, 1.0 kHz, 30 μs	The activation energy of the esterification reaction of ethanol propionate was reduced by 18.9% and the product was 1.9 times higher than that untreated.	(90)
	Fusel oil	Jujube wine	PEF: 1.5 kV/cm, 0.1 kHz, 10 μs	After 140 min treatment, the methanol content decreased by 30.7% compared with the control group.	(43)

#### Promoting extraction of phenolic compounds

Phenolic compounds are important components in determining the quality of fermented wine, which influence the color, mouthfeel, and aging ability of wines. The phenolic compounds in winemaking can be divided into flavonoids (12 major subclasses, mainly anthocyanidins, flavan-3-ols, flavonols, flavanones, isoflavones, and others) and non-flavonoids (mainly hydroxyl cinnamic acid derivatives) (41). The application of PEF in the maceration stage can greatly enhance the content of phenolic compounds in fermented wine. Previous studies have shown that PEF treatments can increase the contents of total phenols, total flavonoids and anthocyanins in wine using different voltages (7–8 kV) and different frequencies (178–344 Hz) (42). The PEF treatment (8 kV/cm, 344 Hz, 300 s pulses) resulted in a wine with a content of total phenols 2 times and 1.5 times higher than the control sample in the case of “Pinot Noir” and “Merlot” red wine, respectively. Apart from phenolic compounds localized in the grape skins, PEF treatment improved the extraction of phenolic compounds that primarily originate from the grape seeds such as flavanols e.g., (+)-catechin. PEF treatment (7.4 kV/cm) on crushed grape berries of Tempranillo and Grenache has also been shown effective in enhancing (+)-catechin and (−)-epicatechin concentrations in the grape juice (37). Particularly, PEF treatment can promote the release of components that are difficult to leach through normal pathways by inducing electroporation of the cell membrane. Arcena et al. (26) observed that the flavonol quercetin along with a few anthocyanins, such as delphinidin-3-O-glucoside and petunidin-3-O-glucoside, were detected exclusively in Merlot musts pre-treated with high energy treatments, which indicated that not all phenolic compounds were easy to be extracted, thus the application of PEF was needed to enhance their extractability. Notably, PEF can also significantly increase the extraction rate of phenols in other fermented wines. Xu et al. (16) took jujube pulp as the research object and carried out different PEF treatments with 10 exponential wave pulses at 1.5 kV/cm, 1 Hz to improve the extraction of phenolic compounds, especially caffeic acid, morin, and p-hydroxybenzoic acid, which significantly strengthened the floral and fruit flavor volatility of jujube wine. In a similar report by Li et al. (43), the phenolic content in the fermented jujube wine after 20 min with PEF (1.5 kV/cm, 10 μs, 0.1 kHz) increased by 45% compared with the control group.

The variation trend of phenolic content was not consistent with different PEF treatment conditions, which is closely related to the variety of raw materials. El Darra et al. (44) applied PEF treatments with medium and high field intensity (0.8 and 5 kV/cm) to improve the total polyphenols content of the pre-treated extract of Cabernet Sauvignon red grapes during low-temperature maceration, showing that color intensity, anthocyanins content, and the extraction kinetics of phenolic compounds were significantly enhanced. Other authors confirmed that the extraction rate of phenols from Cabernet Franc grapes increased by 51 and 62% after PEF treatment (0.8–5 kV/cm, 42–53 kJ/kg), respectively (45). A similar finding was reported by investigating the effect of PEF at different electric field strengths (up to 41.5 kV/cm) and energy inputs (up to 49.4 kJ/kg) on the volatilities and phenolic profiles of “Merlot” grapes at different stages of winemaking (26). PEF treatment increased the contents of anthocyanins, catechin, stilbenes, hydroxycinnamic acid, and hydroxybenzoic acid in the wine after alcoholic and malolactic fermentation. Compared to the untreated group, juice from PEF-treated grapes was found to be at least 7–49 times higher in individual anthocyanins. The stilbenoids, flavonols, and hydroxycinnamic acids at all applied PEF intensities also increased 2–5 times, 2–11 times, and 4–6 times, respectively (26). Interestingly, the content of phenols did not necessarily increase with increasing applied electric field strength. The effect of PEF (0.9–3 kV/cm) on the extractability of polyphenols in early-harvested Sangiovese red grapes from Emilia Romagna (Italy) was investigated by Ricci et al. (46). The results showed that the extraction rate of polyphenols in the PEF-treated groups (10.4, 23.8, 32.5 kJ/kg) increased by 22.9, 16.1, and 20.4%, respectively, compared with the control group. More notably, the total phenolic content of the PEF-treated groups (10.4, 23.8, 32.5 kJ/kg) was significantly increased by 49.0, 60.8, and 60.7%, respectively, after maceration (46). Therefore, when applying a PEF treatment, the transmembrane transport of bioactive compounds was regulated by pore dimensions produced during electroporation and by the size of the passing molecules, which means that the extent of PEF intensity may modulate the composition and amount of the polyphenol components (14).

In general, according to published research results, it is not difficult to find that PEF treatment increases the content of total phenolic compounds and effectively promotes the dissolution of components, and the main influencing factors are electric field intensity, treatment time, and raw material type. Due to the electroporation effect, there are three main trends in the pretreatment of raw materials by PEF. Firstly, the yield of juice during the maceration stage was increased. Secondly, the extraction efficiency of compounds from the skins and the seed of raw materials was improved, which was manifested by the increase of phenolic substances such as hydroxycinnamic acids, malvidin-3-O-glucoside and (+)-catechin. Finally, the higher applied electric field strength may lead to a decrease in the extraction rate of polyphenols, and there is an optimal value of PEF treatment conditions.

#### Decreasing maceration time

**FIGURE 2:**
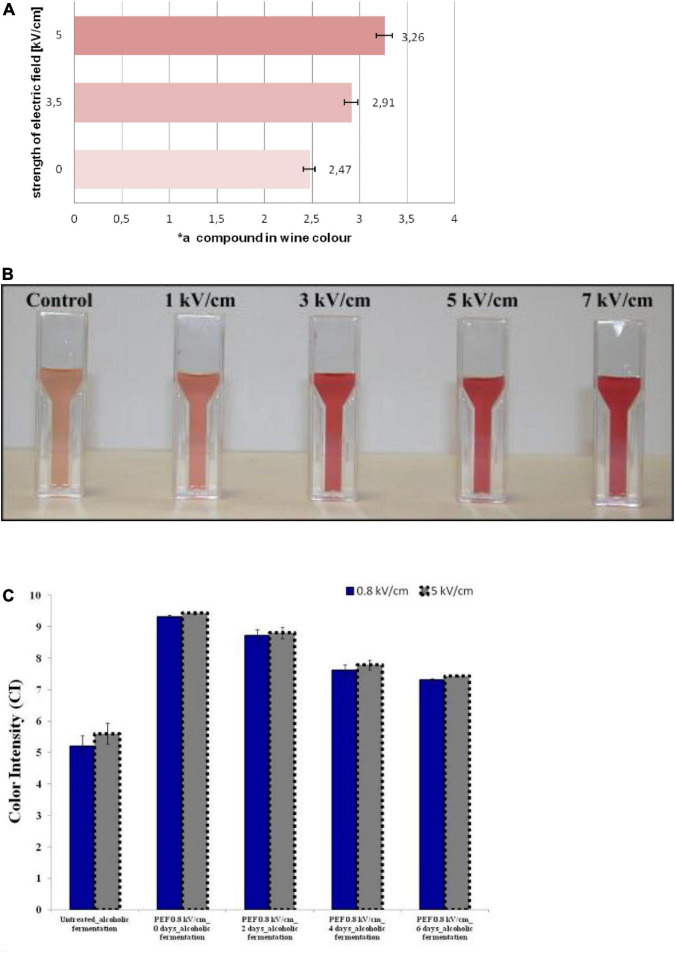
**(A)** Share of the red component (*a) in the color of the control group and PEF treatment (3.3 and 5 kV/cm) wines. Reprinted from Puértolas et al. (48). **(B)** The color of the Garnacha must significantly enhanced with the increase of PEF treatment (1–7 kV/cm, 0.4–4.1 kJ/kg, 50 pulses). Reprinted from Hui et al. (85). **(C)** Color intensity (CI) at *t* = 0, 2, 4, and 6 days of Alcoholic Fermentation using both high (5 kV/cm) and moderate (0.8 kV/cm) PEF. Reprinted from El Darra et al. (44).

The extraction of pigment compounds from the cells of the skin during the maceration step is controlled by diffusion through the cell membranes in the winemaking process (46). The extraction requires that the pigment compounds leave both the membrane-bound vacuole and the cell itself, which is a slow process that requires several days or even a dozen days, while PEF treatment can significantly shorten the maceration time and ensure adequate pigment extraction. The influence of PEF treatment (5 kV/cm, 2.1 kJ/kg) on the grape pomace on quality parameters and anthocyanins content of Cabernet Sauvignon wines obtained after different maceration times has been investigated (47). Regardless of the maceration time, the freshly red wine was richer in color intensity, total polyphenols index, and tannins, and showed better visual characteristics with PEF treatment. Meanwhile, it was detected that the concentrations of malvidin-3-glucoside and malvidin-3-glucoside acetate, as the main anthocyanins in red wine, were higher than those in control wine. Similar results were observed in the study of Puértolas et al. (48), where the maceration time for PEF-wine was only 96 h, 33% less than the control wine that was macerated for 144 h. According to the results obtained, the application of PEF treatment can significantly reduce the maceration time during winemaking. Interestingly, when using a pre-fermentative maceration at 12°C for 6 h, PEF-wine (0.8 and 5 kV/cm) presented an anthocyanin concentration of 36% higher than the control (Figure 2C). In contrast, Ducasse et al. (49) using a maceration scheme consisting of 12 h at a temperature of 15°C, obtained an increment of 13% on the anthocyanin content of Monastrell rosé wines using an enzymatic preparation. This indicated that PEF treatment can effectively improve the extraction rate of anthocyanin than enzyme treatment.

### Enhancement of color

The color of fermented wine is usually an important index to measure its quality. For red wine, its color is generally determined by a mixture of pigment compounds (tannins, anthocyanins, flavonols, etc.), influenced by various factors such as variety, harvest year, grape ripeness, health, winemaking techniques, age, and storage conditions (50, 51). The color was determined using the CIELab method in the produced wines, obtaining L, * a, and * b values, which are the axes of a three-dimensional color space (52). According to research, PEF has a positive effect on the color of red wine, without affecting wine characteristics such as alcohol content, total acidity, pH, sugar concentration, and volatile acidity. As reported in Ilona et al. (53), in the maceration stage, the grapes were treated with a PEF treatment of 3.3 and 5 kV/cm for 10 s (Figure 2A). The share of the red parameter (*a) in wines is respectively higher by 15 and 24% than those of the samples without additional pretreatment (Table 1).

The color of the Garnacha must after maceration significantly enhanced with the increase of PEF treatment conditions (50 exponentially decaying pulses; 1–7 kV/cm, 0.4–4.1 kJ/kg) (Figure 2B). With PEF treatment (5 kV/cm, 122 Hz, 50 pulses) during aging in the bottle, the color intensity and Folin–Ciocalteu index in red wine from Cabernet Sauvignon grapes increased by 31 and 25%, respectively (48). Anthocyanin content in Cabernet Sauvignon rosé wines obtained from grapes treated with PEF (5 kV/cm, 50 pulses) was investigated. After 2 months of storage in bottles, varying extents of increases were observed in fourteen different anthocyanins (unacylated, acylated, and coumarylated) compared to the control group (54). The results showed that the extraction rate of wine-specific pigment compounds was higher under the support of PEF due to the effect of electroporation, which was beneficial because the color of darker wine indicated high levels of colored antioxidant beneficial compounds. Furthermore, the application of PEF on a semi-industrial scale has also achieved satisfactory results. The researchers examined the effect of PEF pretreatment (0.4–7 kV/cm, 100 μs–10 ms) on wine color at a scale of 2 tons per hour (55). Red wine produced by PEF-treated grapes had a 20–30% higher color intensity and a 7–17% higher total polyphenol index than the control group. Surprisingly, the total anthocyanin content of PEF wine was 34% higher than that of the control.

The purplish tones of young red wine typically develop into more stable brick-like colors of matured wines during storage (56–58). During the aging period after fermentation, the proanthocyanidins in wine will self-polymerize and form high molecular weight polymers with anthocyanins. In addition, the flavan-3-ols component unit of proanthocyanidins will form condensates with anthocyanins or acetaldehyde, resulting in the maturation of wine color (59). The content of proanthocyanidins, average degree of polymerization and component units of proanthocyanidins in Merlot red wine changed significantly after high voltage PEF treatment (6–24 kV/cm, 10 μs, 10 Hz, 0–300 pulses), and the trend of change was consistent with the natural aging effect (60). When the electric field strength was lower than 18 kV/cm, the treatment effect was closer to aging with the increase of the field strength. However, it is worth noting that when the field strength was up to 24 kV/cm, the excessive field strength would promote the participation of proanthocyanidin in the chemical reaction, and its degradation rate would exceed the formation rate, leading to the decrease of treatment effect. Therefore, moderate PEF treatment is beneficial to accelerate wine aging and stabilize wine color.

### Inactivation of spoilage microorganisms

**FIGURE 3:**
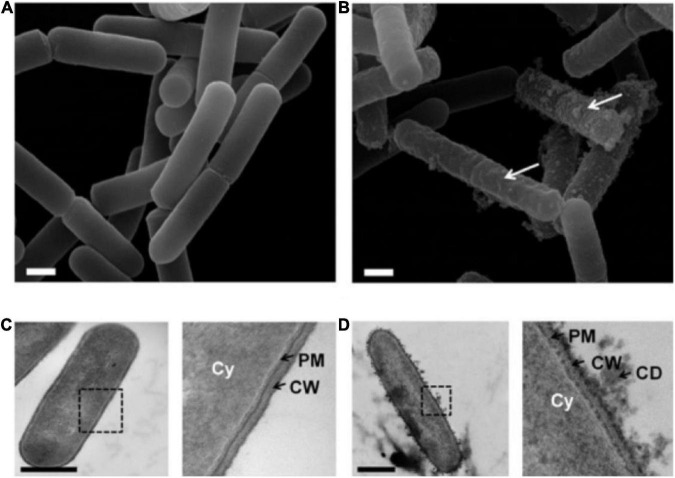
**(A)** SEM image of untreated bacteria. **(B)** After exposure to PEF (7.5 kV/cm, 1 ms), the SEM image showed bacterial surface damage. **(C)** TEM image of untreated *Bacillus pumilus*. Cy, cytoplasm; PM, plasma membrane; CW, cell wall. **(D)** TEM image of *Bacillus pumilus* after PEF. The illustration revealed the discharge of cell debris (CD) and the damage to PM and CW. Scale bars, 500 nm. Reprinted from Davaux et al. (71).

Yeasts and bacteria are common spoilage microorganisms in fermented wine, which negatively affect the quality and shelf life of wine and cause detrimental economic losses (61, 62). SO_2_ is generally used in the traditional winemaking process as an antioxidant and selective antibacterial additive to inhibit the growth of molds in the must during the early stage of wine production and the growth of undesirable bacteria and yeast during the fermentation process, to avoid microbial spoilage in the wine production and storage process (63). The problem is that SO_2_ can cause some adverse effects on consumers including allergic reactions, headaches, asthma, and abdominal pain. Therefore, the addition of SO_2_ is limited to a maximum of 150–350 mg/L. PEF leads to membrane electroporation and even disintegration due to its short and high electric field intensity pulses (Figure 3), which have a significant inactivation effect on microorganisms. Hence, the application of PEF to the microbial inactivation step of fermented wine can reduce or even replace the amount of SO_2_ addition in winemaking, while achieving the expected sterilization effect (64, 65).

#### Lethal effects

Pulsed electric field treatment has proven to be a highly efficient wine pasteurization technique because it can inactivate key spoilage microorganisms in a short time, while retaining the distinctive flavors and aromas of the wines produced, without influencing taste and quality (66). There may be a variety of spoilage yeasts in winemaking, such as *Saccharomyces cerevisiae*, *Saccharomyces bayanus*, *Zygosaccharomyces fermentati*, and species of *Candida, Pichia*, and *Hansenula*, which may lead to the formation of thin-film growth on the surface of the wine, affecting the quality of wine (67) (pp. 535–676). A variety of bacteria can also be present in wine, such as *Lactobacillus brevis, Oenococcus oeni*, *Lactobacillus buchneri*, and *Pediococcus*, which can cause spoilage, pH rise, turbidity, discoloration, stale taste, and sediment formation (67) (pp. 535–676).

The inactivation effect is closely related to the species of microorganisms, due to the different sensitivity of microorganisms to PEF treatment. *S. cerevisiae* can cause a secondary fermentation of the wine when residual sugar is present, eventually forming a precipitate in the wine. Abca and Evrendilek (68) found that a 3 μs square bipolar pulse with a field intensity of 31 kV/cm resulted in 4.5 log reductions of *S. cerevisiae* in red wine. When the same electric field intensity was applied to *S. bayanus* in red wine, the inactivation rate was significantly increased, reaching 5.4 log reductions. The PEF resistance of different wine spoilage microorganisms such as *Dekkera anomala, Dekkera bruxellensis, Lactobacillus hilgardii*, and *Lactobacillus plantarum* both must and wine was investigated by applying treatments ranging from 16 to 31 kV/cm and from 10 to 350 kJ/kg at 24°C (69). The optimal treatment with a specific energy of 186 kJ/kg has been established which permitted to reduce the 99.9% of the spoilage flora of must and wine at the field strength of 29 kV/cm, limiting the risk of alteration of these products by microorganisms of genera *Brettanomyces* and *Lactobacillus*. Concerning bacteria in wine, Gonzalez-Arenzana et al. (65) treated 12 lactic acid bacteria and 4 acetic acid bacteria in Tempranillo red wine under four different PEF conditions with specific energies of 60, 62, 72, and 95 kJ/kg, respectively. The inactivation rate was around 1.93 log reductions (*Pediococcus pentosaceus*) and 3.56 log reductions (*Acetobacter pasteurianus*) at PEF treatment of 62 kJ/kg (22 kV/cm, 154 μs), (Table 1). The inactivation cycles obtained with the 72 kJ/kg PEF treatment (27 kV/cm, 123 μs), were between 0.64 log reductions (*Acetobacter aceti*) and 2.44 log reductions (*Metschnikowia pulcherrima*). The 95 kJ/kg PEF treatment (33 kV/cm, 105 μs) managed inactivations between 1.96 log reductions (*O. oeni* strain O46) and 4.94 log reductions (*A. pasteurianus*).

A similar effect was achieved with PEF treatment in rice wine. Lyu et al. (77) found that 2.88 log reductions of *Saccharomyces cerevisiae* were inactivated when PEF treatment with moderate conditions (35°C, 12 kV/cm, 120 μs) was applied to Chinese rice wine. Further, Huang et al. (70) used PEF technology to evaluate the inactivation effect of *S. cerevisiae* at the initial temperature of 25–35°C, electric field intensity of 12–21 kV/cm and treatment time of 30–180 μs. The highest inactivation values, corresponding to approximately 5.5 log reductions, were obtained at 21 kV/cm and 180 μs with the initial treatment temperatures of 35°C. Davaux et al. (71) investigated the effects of the PEF (7 kV/cm) on the microbiological stability of wines on a semi-industrial scale from 500 to 1,200 L/h. When the temperature of wine without adding SO_2_ did not exceed 50°C, PEF had a significant inactivation effect on yeast in wine (71). The results obtained showed an excellent efficiency of the yeast treatment with an instant cessation of alcoholic fermentation and a decrease in the yeast population ranging from 3 to 5 log reduction.

#### Sublethal effects

Recently, the sublethal effects of PEF on microorganisms have attracted more and more attention. There are many factors affecting the microbial inactivation by PEF and one of the key contributing factors is critical electric field intensity, which is defined as the minimum electric field intensity required for microbial inactivation. Previous studies have shown that the intensity of the applied electric field must exceed a critical electric field intensity for electroporation (reversible or irreversible) of the cell membrane to occur (72, 73). Notably, when the cell membrane undergoes reversible electroporation after PEF treatment, a sublethal state of the cells may be induced (73). In general, the critical electric field strength value depends on microbial characteristics, especially on cell size and shape, and the characteristics of the growing medium (72). Critical electric field intensity was lowest for the yeast (14.83 kV/cm) and it was 18.64 kV/cm for the aerobic bacteria (73). Whereas, the highest critical electric field intensity value of 19.46 kV/cm was noted for the lactic acid bacteria (73). This variation in PEF sensitivity of the microorganisms could be due to the differences in cell size since the relatively large-sized cells (yeast) inactivated at the lowest electric field intensity. Generally, with the increase of voltage amplitude, capacitance capacity, and discharge times, the bactericidal rate keeps improving. When the injection energy is more than 12 J/ml at normal temperature, the bactericidal rate of PEF (15–25 kV/cm) is more than 97%, and the maximum 2-log reduction inactivation rate can be achieved (74).

*Brettanomyces bruxellensis* is mainly found in barrel-aged red wines with low SO_2_ content and high pH (75). The researchers inoculated the wine with *B. bruxellensis* and then treated it with continuous PEF. The concentration of *B. bruxellensis* in wine was determined after inoculation, ranging from 2.3 × 10^5^ to 5.9 × 10^5^ CFU/ml. The *B. bruxellensis* concentration decreased to 9.2 × 10^4^ CFU/ml (0.8 log reduction) after PEF treatment (32 kV/cm, 250 Hz, 51.2 μs). However, the yeast concentration had returned to the same level as the untreated wine after 2 months. Therefore, moderate PEF treatment conditions which could have induced a sublethal state instead of cell death, are not sufficient to inactivate *B. bruxellensis* to produce microbiologically stable wines (66). To completely inactivate *B. bruxellensis* in wine, more demanding PEF conditions are required, including higher electric field intensities and longer treatment times. A recent study found that multiple PEF treatments (50 kV/cm, 100 Hz, 78 μs) achieved the expected level of *B. bruxellensis* inactivation in Cabernet Sauvignon red wine (>6 log reductions) (25).

It is worth noting that the efficiency of PEF inactivating microorganisms is also closely related to food medium (processed raw materials) factors such as alcohol and temperature. For example, *Acetobacter* sp. is also one of the spoilage microorganisms during winemaking, converting ethanol produced by yeast into acetic acid, which increases the volatile acidity of the wine. The effect of ethanol as a growth substrate on PEF resistance in *Acetobacter* sp. cells was investigated by Niu et al. (22). The inactivation rate of *Acetobacter* sp. (10^9^ CFU/ml) cultivated with 9% ethanol by PEF treatment (20.0 kV/cm, 6.0 ms) could reach 5.17 log reductions, which was significantly higher than that cultivated without ethanol (3.22 log). According to the report, the combination of eugenol and PEF has a strong synergistic bactericidal effect. The inactivation level of *Escherichia coli* was increased from 0.39 to 0.96 log when the added eugenol concentration was increased from 0 to 0.64 mg/ml and PEF treatment intensity was 20.0 kV/cm (76). Besides, increasing the initial temperature of fermented wine during PEF treatment had a significant effect on microbial inactivation. The survival yeast decreased with 4.09 log reductions when the Chinese rice wine was preheated to 35°C and followed by PEF treatment (18 kV/cm, 150 μs). In comparison, the yeast inactivation was 2.05 log reductions at an initial temperature of 25°C and the same PEF treatment (77).

In summary, PEF used for microbial inactivation can maximize the sterilization effect, inactivate enzymes, protect the nutritional components in wine, and require low processing temperature, a short processing time of several decades microsecond to sterilize. However, it was very difficult to summarize and systematically classify resistance to PEF based on size, shape or membrane structure due to the high number of different microorganisms. The inactivation of microorganisms associated with fermented wine depends on the PEF conditions applied and the characteristics of the microorganisms (78). The diversity of microbial species which are PEF resistant indicates PEF sterilization needs strict verification before application.

### Formation of aroma substances during aging

**FIGURE 4:**
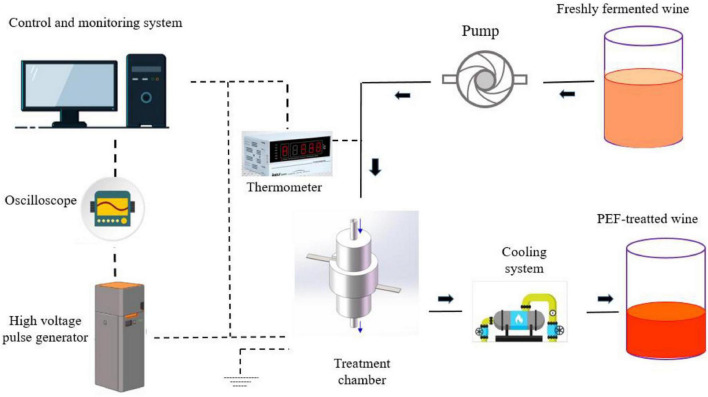
Schematics diagram of a PEF treatment system for fermented wines. Adapted from Wang et al. (10).

**FIGURE 5:**
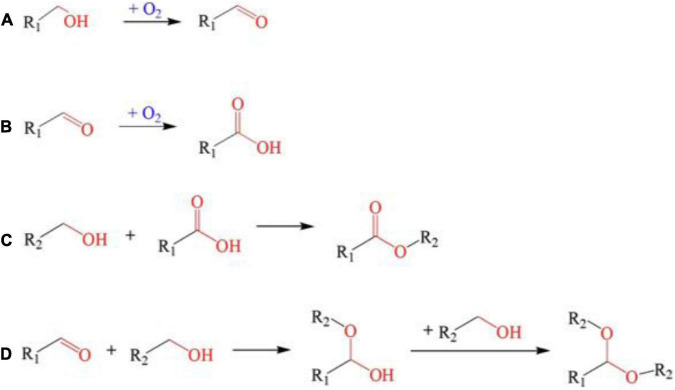
The reactions promoted by PEF treatment. **(A)** Alcohols oxidized into aldehydes; **(B)** aldehydes oxidized to acids; **(C)** Esterification reaction; **(D)** Polymerization reaction of alcohols and aldehydes could condensate into acetal. Reprinted from Ma et al. (97).

Freshly fermented wines usually have some disadvantages such as sourness, thin taste, stimulating taste, and many other negative impacts due to the high content of fusel oil, acetal, and free alcohol. Hence, aging is needed to stabilize the wine quality, while various biological and chemical reactions occur during the aging process, resulting in complex aromas and full-bodied mouthfeel (79). It is reported that high-intensity PEF treatment (usually >15 kV/cm) can accelerate the chemical reaction rate in dynamic equilibrium and reduce the activation energy required for the reactions (Figures 4, 5), which can significantly promote the formation of various aroma components such as thiol, acids, polyphenols, and esters in fermented wine (28). For example, it was found that Merlot red wine vinified with PEF-treated grapes possessed higher thiol concentrations and higher intensities of blackcurrant flavor character at different electric field strengths (up to 41.5 kV/cm) and energy inputs (up to 49.4 kJ/kg) (26).

#### Fatty acids

Moderate PEF markedly increased the fatty acid content of fermented wine because it enhanced the oxidation of ethanol to aldehydes and further to acids (80). Different moderate PEF treatments (6–24 kV/cm) and pulse numbers (100–300 pulses) with red wine resulted in the evolution of L-malic acid, citric acid, and succinic acid concentrations following similar trends to bottle aging (10). The variation trends of citric acid, oxalic acid, and succinic acid in naturally aged white wines, were similar to those of white wines treated by PEF (6 kV/cm, 100 pulses) (Table 1). The amount of L-malic acid in the wine treated by PEF of 6 kV/cm and 100 pulse numbers was near to that of bottle storage for 90 days. The phenomenon revealed that the application of external PEF affected the molecular structure and chemical reactions among the substances in wine (81). Similarly, the fatty acids such as isovaleric acid, hexanoic acid, and octanoic acid in Chinese rice wine treated with PEF (2 kV/cm, 0.5 Hz, 36 pulses) could reach the level of natural aging for 3 years (82).

It is noteworthy that the variation trend of different fatty acids after PEF treatment was significantly different. For example, PEF was able to decline the content of tartaric acid in white wine and then decreased more steeply with a further increment of PEF strength. This trend is consistent with the change in bottle storage and may be related to chemical reactions between tartaric acid and other components. High levels of PEF allow the rapid evolution of tartaric acids, forming caffeoyltartaric acid, and ρ-coumaroyl tartaric acid (10). Furthermore, high-intensity PEF may have some negative effects on the changes of fatty acids in fermented wine. Succinic acid, for example, is created as a by-product of the sucrose fermentation process in wine and enables the taster to perceive the acidity, bitterness and acidity of wine in high concentrations (83). Zeng et al. (81) reported that the succinic acid content was lower than that of the untreated group due to the conversion of succinate to ethyl succinate after PEF treatment at a lower intensity. However, high-intensity PEF treatment at 18 and 24 kV/cm significantly promoted the hydrolysis of diethyl succinate, and the content of succinic acid increased (10). Therefore, the change in fatty acids depends on the strength of PEF, and there may be an optimal treatment condition for different acids.

#### Free amino acids

The taste characteristics of free amino acids can be divided into several categories, which were MSG-like (Asp and Glu), sweet (Ala, Gly, Ser, and Thr), and bitter (Arg, His, Ile, Leu, Met, Phe, and Val), based on the study of Azevedo et al. (84). The sweet and MSG-like components of Shaoxing Huangjiu (Chinese rice wine) treated with PEF (2 kV/cm, 0.5 Hz, 36 pulses) could reach the level of aging for 3 years (82). In terms of bitter amino acids, the contents of Huangjiu treated with accelerated aging showed a general trend of decline (Table 1). Compared with the naturally aged Huangjiu, PEF treatment reduced the bitterness of the wine and tempered the natural flavor of Huangjiu. This result was consistent with the research of Xu et al. (16), which found that Asp, Glu, Gly, and Ser increased after PEF treatment (1.5 kV/cm, 1 Hz, 50 pulses) in jujube wine. Free amino acids are precursors of flavor compounds (11), these free amino acids may be highly correlated to the complex synthesis of flavor compounds, which is of great significance to the overall aroma of Chinese rice wine. It is worth noting that high-intensity PEF treatment may promote the Maillard reaction between sugar and amino acids in rice wine, thus reducing the content of bitter-type amino acids (44, 84, 85).

#### Polyphenols

The content of polyphenols in fermented wine after PEF treatment was increased, such as flavan-3-ols, flavonols, hydroxycinnamic acids, and their derivatives, which is due to the high concentration of precursors in PEF-wine (86). For example, high concentrations of tartrate esters are hydrolyzed into their corresponding free acids such as caffeic acid and coumaric acids, leading to an increase in hydroxyl cinnamic acid. The surge in hydroxybenzoic acids, particularly gallic acid, protocatechuic acid, and syringic acid, may have resulted from the cleavage of anthocyanins during wine storage (e.g., malvidin 3-O-glucoside may lead to the production of syringic acid) (87). However, PEF treatment may also lead to a reduction in the evolution of some specific phenolic substances during aging. For red wines treated with PEF at specific energy (49.40 kJ/kg), prolonged storage at 4°C significantly reduced the amount of 11 Phenolic compounds, including anthocyanins, flavonols, and stilbenes (80). As mentioned earlier, this reduction may be due to degradation, oxidation, and cleavage auto association, as well as co-pigmentation, polymerization, and formation of new pigments (88). Due to the high initial anthocyanin content throughout the winemaking process, the level is still above that of naturally aged wines, despite the loss of large amounts of anthocyanin during storage. Moreover, the condensation reaction between (+)-catechin and acetaldehyde was enhanced by the PEF treatment (Figure 5). With the increase of PEF intensity ranging from 0 to 50 kV/cm, the decrease rate of (+)-catechin increased obviously. It was reported that at 40 kV/cm, the content of (+)-catechin after 31.12 ms reaction was roughly equivalent to the control group for 62.23 ms reaction without PEF treatment. Meanwhile, PEF treatment could significantly reduce the activation energy of the condensation reaction from 41.59 to 28.98 kJ/mol (89). Similarly, polyphenol flavan-3-ol monomers catechin and epicatechin promoted polymerization to form tannins under PEF treatment (4 kV/cm, 100 μs, 6.2 kJ/kg), which was similar to naturally aged Grenache wines (13).

#### Esters composition

Esters are the main source of aroma in wine (90) and PEF treatment can promote the esterification reaction in fermented wine (Table 1). The reaction rate and product concentration of the esterification reaction increase with the increase of electric field intensity. The effect of PEF (0–35 kV/cm, 1.0 kHz, 30 μs) on ethanol esterification of propionate was investigated by Lin et al. (91), while ethyl propionate had a distinctive fruity aroma. The content of ethyl propionate produced by PEF treatment was 1.30, 1.80, and 1.9 times higher than that without PEF treatment when the reaction temperature was 20°C and the field strength was 6.6, 13.3, and 20 kV/cm, respectively. Similarly, ethyl lactate possessed a distinctive rum, fruit and cream aroma. When 20 kV/cm PEF was applied and the reaction temperature was 25, 30, and 35°C, the yield of ethyl lactate was 1.7, 1.8, and 1.4 times of the sample without PEF (92). In addition, PEF can reduce the activation energy of esterification under certain conditions. When the electric field intensity is 13.3 kV/cm, the activation energy of esterification is 62.85 kJ/mol, which is 18.4% lower than the 77.05 kJ/mol of the control sample. In practical application, Shen et al. (82) treated Chinese rice wine samples with PEF (2 kV/cm, 0.5 Hz, 36 pulses) to explore the effect of PEF on the aroma substances of rice wine. The oxidation and esterification reaction could be promoted and the ethanol content would be greatly reduced in the PEF-treated Chinese rice wine. Fatty acid ethyl esters treated with PEF (2 kV/cm) could reach the level of natural aging for 3 years, especially in medium-chain (C6–C12) and long-chain (C13–C18) fatty acid ethyl esters. Among them, ethyl 2-hydroxypropionate, ethyl butyrate, ethyl valerate, and ethyl palmitate increased most significantly (82). The technical principle of PEF promoting aging is to use the energy provided by high-voltage to change the wine body into a strong oxidation state and accelerate a series of reaction processes such as oxidation-reduction and esterification (Figure 5).

#### Unpleasant substances

Pulsed electric field treatment can reduce the concentration of some irritant volatile substances below the reported odor threshold, potentially having a positive effect on overall wine perception. The fatty acids such as octanoic acid, capric acid, butyric acid, and 3-methylbutyric acid significantly decreased in the presence of PEF (11, 22 kJ/kg) (14). The concentrations of the last three compounds were lower than the odor threshold (15, 10, and 3 mg/L, respectively) after PEF treatment, which could cause a cheese-like, rancid and pungent odor at high concentrations (93). In addition to fatty acids, the concentrations of some volatile phenols (such as 4-vinylphenol and 4-vinylguaiacol) were also significantly reduced by PEF processing. The presence of these two compounds in white wine and red wine is due to enzymatic decarboxylation of cinnamic acids by yeasts and is generally considered a defect in wine because they have unpleasant smells similar to drugs and paints (94). Most notably, excessive fusel oils which are a mixture of higher alcohols in freshly wine will make wine bitter, spicy, and rough taste. Appropriate high voltage PEF treatment (5–20 kV/cm) can promote the oxidation of fusel oil in wine, so that it can transform into aldehydes or acids, resulting in a decrease and corresponding reduction of fresh wine irritation (95). Similarly, some researchers pretreated jujube pulp with PEF (1.5 kV/cm, 10 μs, 0.1 kHz) and found that within a certain range, along with the increase of PEF treatment time, the methanol content in the fermented jujube wine decreased significantly, and the maximum reduction reached 30.7% compared with the control group after 140 min of treatment (43).

## Challenges and future trends

In previous studies, the potential of PEF in the raw material extraction, sterilization and aging of fermented wine has been extensively explored in laboratory studies, and gradually applied in pilot-scale and semi-industrial production. Nevertheless, further investigations should be carried out for some controversial results about the application of PEF in fermented wine.

For commercial application, PEF needs to achieve breakthroughs in the core components of equipment such as high-power supply and intelligent control systems. The focus should be on the applicability of PEF for broad-spectrum processing of different materials such as high conductivity or high viscosity, the uniformity under the action of the physical field, and the controllability of the processing temperature. At the same time, the stability, processing capacity and production efficiency of PEF system should be greatly improved to realize the large-scale application, continuous processing and intelligent control of PEF in the fermented wine industry. Moreover, since the electrode is in direct contact with the wine, the electrochemical reaction at the interface may cause corrosion and migration of the electrode material, resulting in the flavor of the PEF-treated wine being affected. Some researchers even tasted metallic in some PEF-treated samples (15). Therefore, it is necessary to develop more suitable or more durable electrode materials to replace the commonly used stainless steel electrode.

On the other hand, the principle of accelerated aging of fermented wine after PEF treatment and the optimal application parameters of PEF are still unclear. The complex reactions of multiple compounds occur under the action of PEF, and flavor in wine is affected by the combination of all the compounds, which means that some trace substances may have a unique effect on flavor. It is difficult to capture the effects of trace substances in conventional analysis methods, making it difficult for researchers to detect subtle changes caused by PEF. More importantly, for specific compounds, such as phenols, alcohols, acids, esters, and other aroma components, the effect of PEF is not all positive. The effects of PEF on some desired substances have disparate trends for different fermented wines. However, most current research on PEF mainly focus on the application of pre-maceration and the evolution of phenolic components during red wine aging while other kinds of fermented wine are rarely reported. Furthermore, various aging conditions such as container material and aging temperature, give the wine a kaleidoscope of styles during aging. PEF treatment alone cannot completely achieve the complex aroma and taste of the natural aging process. Therefore, combining PEF technology with other processing technologies such as ultrasound, microwave, and micro-oxygenation to obtain fermented wines that are closer to natural aging may be a direction worth exploring.

## Conclusion

Research on the applicability of PEF in the fermented wine industry suggests that PEF can not only shorten the maceration time of brewing raw materials and promote the extraction of main functional components, but also enhance the color of fermented wines, inactivate spoilage microorganisms, and accelerate the formation of aroma substances during the aging process. Additionally, appropriate PEF treatment can reduce the levels of some unpleasant substances, especially fusel oil in freshly fermented wines. Furthermore, some laboratory and semi-industrial studies on the application of PEF technology have achieved the expected results. However, it is worth noting that there are still some bottlenecks that need to be solved urgently, such as the development of corrosion-resistant electrodes, high-power supplies, and intelligent control systems, which hinder the large-scale industrial application of PEF technology in the fermented wine industry.

## Author contributions

YF: writing—original draft, software, formal analysis, and visualization. TY: writing—review and editing and validation. YZ: conceptualization and data curation. AZ: investigation and resources. LG: writing—review and editing and investigation. DN: funding acquisition, supervision, writing—review and editing, resources, and project administration. All authors contributed to the article and approved the submitted version.
